# Quantitative Investigation of the Fate and Behavior of Antimony Micro/Nanoparticles in Simulated Body Fluids

**DOI:** 10.3390/nano16160974

**Published:** 2026-08-07

**Authors:** Yujian Lai, Sujuan Yu, Zhensong Zhang, Lijie Dong

**Affiliations:** 1 Department of Environmental Science and Engineering, School of Energy and Environmental Engineering, The University of Science and Technology Beijing, Beijing 100083, China; 2 Research Center for Eco-Environmental Sciences, Chinese Academy of Sciences, Beijing 100085, China; 3 Division of Chemical Metrology and Analytical Science, National Institute of Metrology, Beijing 100029, China

**Keywords:** antimony micro/nanoparticles, dissolved form, simulated body fluid, quantification, transformation behavior

## Abstract

Antimony micro/nanoparticles (Sb MNPs) are key environmental Sb species that enter the human body via inhalation, ingestion, and dermal contact, posing potential health risks. Their complex transformation across multiple Sb species hinders accurate quantification, leaving their in vivo transformation mechanisms poorly understood. In this study, in vitro respiratory, gastrointestinal, and sweat models were established to investigate Sb MNP biotransformation, and the gastrointestinal model incorporated human fecal suspension to better mimic in vivo conditions. Transformation dynamics showed that simulated gastric fluid dissolved Sb_2_O_3_, Sb_2_S_3_ and Sb_2_O_5_ MNPs into ionic Sb without altering valence states, while Sb ions remained at low levels. Gastric-derived Sb(III) was oxidized to less toxic Sb(V) in the intestinal phase. In simulated sweat, Sb(III) concentrations increased but accounted for only 1.75% of total exposure, indicating low dermal risk. Notably, Sb_2_O_3_ MNPs exhibited lability in simulated lung fluids, particularly artificial lysosomal fluid (ALF), where dissolved Sb(III) reached 1276.7 μg/L with an ionic release rate of 63.8%. The low pH of ALF and formation of stable soluble complexes with citrate, lactate, and Cl^−^ might drive Sb_2_O_3_ dissolution, suggesting high inhalation risk. This study advances the quantitative understanding of Sb MNP biotransformation and thus for human health risk assessment.

## 1. Introduction

Due to both natural and anthropogenic sources, antimony and its compounds are widely distributed across soil, water, the atmosphere, and other environmental media [1]. Natural weathering of antimony ores, large-scale mining operations, and the production, use, and disposal of antimony-containing products release antimony into the environment. In addition, it undergoes long-range transport via atmospheric dispersion, dry and wet deposition, and fluvial transport, leading to the global spread of antimony contamination. Antimony micro- and nanoparticles (Sb MNPs) constitute a key occurrence form of antimony in the environment. High concentrations of Sb MNPs have been detected in atmospheric particulate matter, primarily originating from anthropogenic activities including coal combustion, metal smelting, and brake pad wear [2,3,4,5,6]. Airborne Sb MNPs can enter the human body through inhalation and dermal contact, resulting in chronic exposure.

Beyond the atmosphere, Sb MNPs are also widespread in soil and aquatic systems, threatening human health via ingestion pathways such as the food chain. Sb MNPs in soil are mainly derived from atmospheric deposition and the weathering of natural antimony ores (e.g., Sb_2_S_3_) [7,8]. In aquatic environments, Sb MNPs partially originate from soil runoff and erosion. Sb MNP dissolution is usually very slow. For example, Sb_2_O_3_ has a half-life of 50–250 days, which allows the particulate antimony to persist for extended periods [9]. Previous studies have reported particulate antimony concentrations as high as 134 ng/L in coastal seawater [10]. Overall, antimony mainly present in particle-associated forms in the environment has natural concentrations of 0.2–32 ng/m^3^ in air [11], 0.3–8.6 mg/kg in soils [12] and 0.1–0.2 μg/L in surface waters [11], which can be elevated by orders of magnitude in anthropogenically disturbed areas such as mining sites and urban zones [13]. Furthermore, Sb MNPs can be taken up by plants and aquatic organisms, thereby entering the human food chain [14,15,16,17]. Meanwhile, synthetic Sb MNPs are extensively used in industrial production and consumer goods. For example, they are added to plastics as flame retardants, applied as catalysts in polyester manufacturing, and used as pigments in glass and ceramics, among other applications [18,19,20]. These products release Sb MNPs during their service life, posing potential human health risks through multiple exposure routes including ingestion, inhalation, and dermal contact.

Antimony and its compounds are genotoxic and potentially carcinogenic. As early as 1979, antimony was designated a priority pollutant by the U.S. Environmental Protection Agency (EPA) [21], and it is also classified as hazardous waste under the EU Basel Convention [22]. The toxicity of dissolved antimony is strongly valence-dependent, with Sb(III) exhibiting approximately 10-fold higher toxicity than Sb(V), the dominant species in oxic environments [23]. The World Health Organization (WHO) guideline value for Sb in drinking water is 20 μg/L, derived from a tolerable daily intake (TDI) of 6 μg/kg body weight/day based on a subchronic NOAEL of 6.0 mg Sb/kg bw/day with a 1000-fold uncertainty factor [11]. More recently, Sb_2_O_3_, with well-documented carcinogenicity in animal models, was added to the list of substances “reasonably anticipated to be human carcinogens” by the U.S. Department of Health and Human Services in 2021 [24]. Sb MNPs exert toxicity through multiple mechanisms: they enter cells via the “Trojan horse” effect [25] and dissolve intracellularly to release high-valence antimony ions, driving a sharp rise in cytotoxicity; they also form a protein corona upon interaction with proteins, which enhances their transmembrane transport efficiency [26]. Furthermore, Sb MNPs trigger oxidative stress by inducing reactive oxygen species (ROS) production, resulting in cell membrane damage, protein denaturation, and DNA damage [27]. Accordingly, systematic evaluation of the human health risks posed by Sb MNPs is critically important.

However, upon entering the human body, Sb MNPs undergo complex physicochemical transformations including agglomeration, ion release, reprecipitation, and Sb(III)/Sb(V) redox-mediated valence shifts [28,29], with transformed species differing markedly in their toxic potency to humans. It is therefore essential to elucidate the transformation behavior and underlying processes of Sb MNPs in the human body. These transformation processes are, however, regulated by a range of physiological factors such as gastric acid, endogenous enzymes, and gut microbiota, giving rise to diverse products and variable reaction kinetics and thus creating major analytical challenges. Accordingly, current research has predominantly focused on dissolved or organically bound antimony fractions [23], while studies on the transformation behavior and species distribution of Sb MNPs in the human body remain scarce. On the other hand, although the metabolism and toxicity of antimony have been explored in model organisms including rats and fish [17,30,31], such models differ from humans in physiological architecture and matrix composition. Furthermore, they fail to enable continuous monitoring of transformation processes, and findings are prone to bias from individual variation. Meanwhile, in vivo studies are also limited by ethical constraints, financial costs, and long experimental timelines.

In vitro simulation approaches offer an effective means to overcome these bottlenecks. By mimicking the fluid environments of the human respiratory, gastrointestinal, and dermal exposure pathways, in vitro models have been developed to investigate the biotransformation of contaminants ranging from dust and fine particulate matter to nanomaterials [32,33,34]. To the best of our knowledge, very limited in vitro transformation studies focusing specifically on Sb MNPs have been reported to date. Compared with conventional particulate matter, the transformation of Sb MNPs is more susceptible to factors including ambient oxygen levels and gut microbiota, yet these critical variables are often not fully incorporated into existing model systems. Accordingly, developing dedicated in vitro models for Sb MNPs that recapitulate complex physiological environments and quantitatively elucidate their transformation pathways, kinetics, and underlying mechanisms represents a pressing scientific challenge. Notably, the in vitro simulation approach has inherent limitations that should be explicitly acknowledged. The simulated biological fluids cannot fully reproduce the complex physiological environment in the human body. Specifically, interindividual variabilities in pH levels, microbiota composition, enzyme activity, and biological secretions may affect the transformation process of Sb particles in vivo.

This study aims to develop in vitro models recapitulating the fluid environments of the human respiratory tract, gastrointestinal tract, and dermal sweat, and to quantitatively characterize the transformation behaviors of typical Sb MNPs (Sb_2_O_3_, Sb_2_S_3_, and Sb_2_O_5_) and antimony ions of varying oxidation states in these systems. Using coupled liquid chromatography coupled with inductively coupled plasma mass spectrometry (LC-ICP-MS) and single particle–inductively coupled plasma mass spectroscopy (sp-ICP-MS) techniques, we dynamically monitor the transformation processes and species distribution of diverse antimony forms in simulated digestive fluids, sweat, and lung fluids, to elucidate their transformation patterns and provide key scientific evidence for the accurate assessment of human health risks posed by Sb MNPs.

## 2. Materials and Methods

### 2.1. Chemicals and Materials

Potassium pyroantimonate (AR, 99.0%), ammonium tartrate dibasic (AR, 99.0%), potassium antimony tartrate (AR, 99.5%), antimony sulfide (Sb_2_S_3_, 98%), antimony(V) oxide (Sb_2_O_5_, 99.95%), antimony oxide (Sb_2_O_3_, 99.99%), and diammonium ethylenediaminetetraacetate monohydrate (98%) were all purchased from Shanghai Macklin Biochemical Technology Co., Ltd. (Shanghai, China). In addition, all glass and plastic containers, tubing, buffers, and biological matrices used for the preparation of simulated biological fluids were sterilized at 121 °C for 20 min prior to use.

### 2.2. Characterization of Sb MNPs

The three types of Sb MNPs (Sb_2_O_3_, Sb_2_O_5_, and Sb_2_S_3_) used in the experiments were characterized via high-resolution transmission electron microscopy (HR-TEM) (JEM-2100F, JEOL, Tokyo, Japan). Energy-dispersive X-ray spectroscopy (EDS) mapping was additionally performed to determine the elemental composition of the particulate samples. For sample preparation, approximately 5 μL of the sample suspension was deposited onto a carbon-coated copper grid and dried overnight under vacuum at room temperature.

### 2.3. Separation and Quantification of Sb Species

Free ionic Sb was separated from the sample matrix by filtration through a 0.22 μm membrane filter, with the ionic fraction recovered in the filtrate. For ionic Sb speciation, Sb(III) and Sb(V) were baseline-separated by liquid chromatography coupled with LC-ICP-MS using the mixture of 25 mM sodium tartrate, 10 mM EDTA, and 80 mM NH_4_NO_3_ as the mobile phase. Detailed instrumental operating conditions are listed in Table S1. For particulate Sb quantification, free Sb species in the samples were extracted into the supernatant via ultrasound-assisted extraction. The number concentration of free particulate Sb in the supernatant was subsequently determined using sp-ICP-MS, with detailed instrumental parameters provided in Table S2.

### 2.4. Development of In Vitro Models

Three in vitro models for gastrointestinal, respiratory, and dermal sweat exposure were developed to correspond to the three exposure pathways investigated in this study. Detailed protocols for model construction and simulated body fluid preparation are provided in Supplementary Materials Text S1–S3.

### 2.5. Exposure of Sb Species in Simulated Body Fluids

To investigate whether Sb MNPs release more toxic free ionic Sb following exposure via the three pathways described above, the analytical methods detailed above were applied to quantitatively monitor the transport and transformation of target Sb species in individual simulated body fluids and across the full gastrointestinal system. For kinetic transformation experiments in individual simulated fluids, Sb_2_O_3_ which was the most widely used yet toxicologically potent Sb compound, was selected as the exposure target. Sb_2_O_3_ was spiked into each fluid at a final concentration of 2 mg Sb/L. Samples were collected at incubation time points of 0.5, 3, 6, 12, 24, and 48 h to track transformation kinetics in simulated saliva (SS), simulated gastric fluid (SGF), simulated small intestinal fluid (SSIF), simulated colonic fluid (SCF), simulated lung fluid, and simulated sweat. For studies examining the transformation and metabolism of diverse Sb species across the entire gastrointestinal system, three Sb MNPs (Sb_2_O_3_, Sb_2_S_3_, Sb_2_O_5_) and two ionic Sb species (Sb(III) and Sb(V)) were individually spiked into simulated saliva at 2 mg Sb/L. After 24 h of sequential incubation through the digestive system, samples were retrieved from each simulated digestive compartment to monitor the transformation behavior of target species along the digestive tract.

## 3. Results and Discussion

### 3.1. Quantitative Analysis of Sb MNPs, Sb(III) and Sb(V)

**Figure 1:**
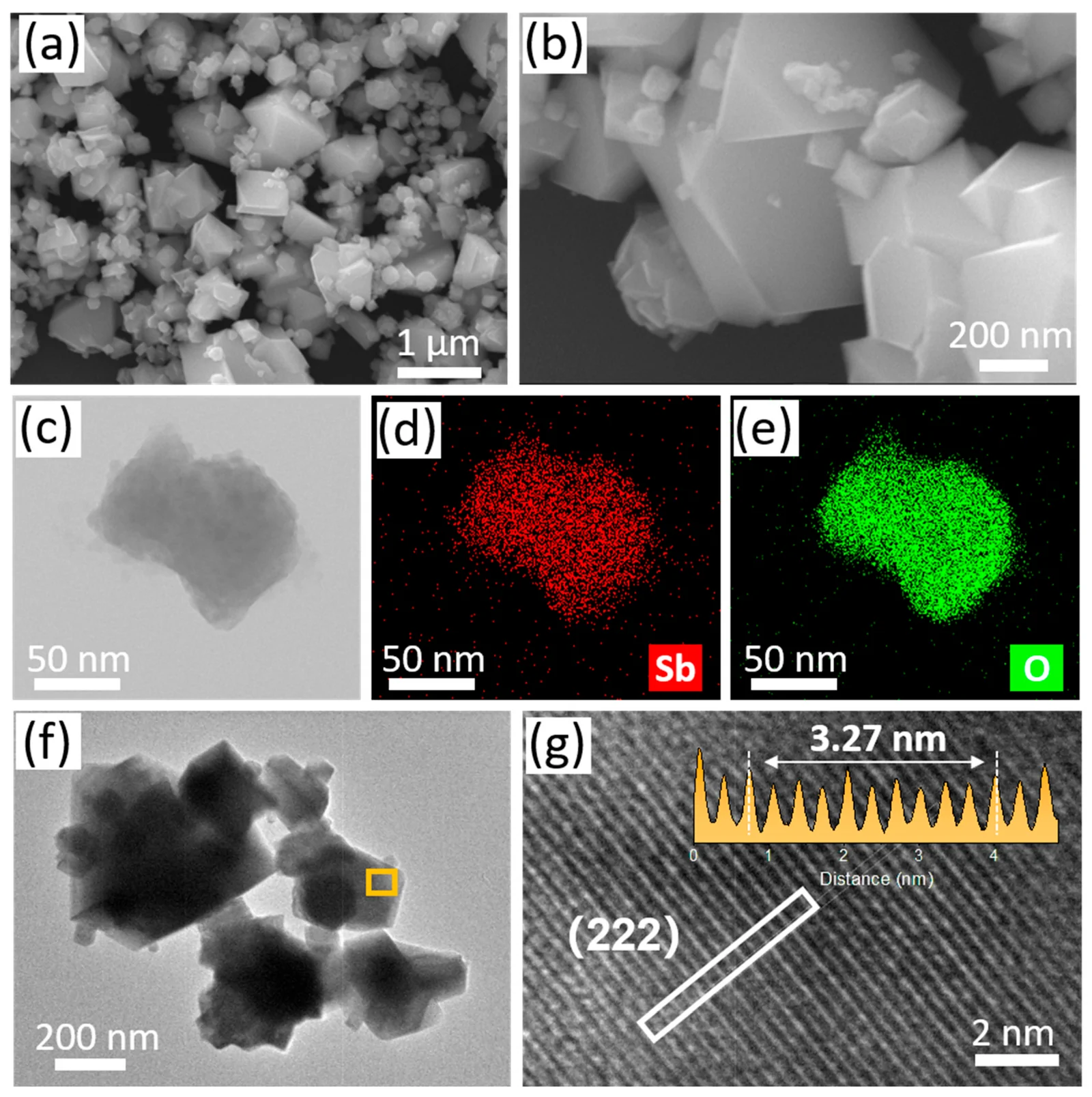
Characterization of Sb_2_O_3_ MNPs. (**a**) Low-magnification SEM image; (**b**) high-magnification SEM image; (**c**) STEM image; (**d**,**e**) EDS elemental mapping images of Sb and O, respectively; (**f**) HRTEM image; (**g**) lattice fringes from HRTEM characterization in the rectangular area of figure (**f**).

Given the anthropogenic and natural origins of Sb MNPs, Sb_2_O_3_, Sb_2_O_5_, and Sb_2_S_3_ were selected as representative species in this study to develop analytical protocols and investigate their transformation behavior in simulated human biological media. Notably, Sb_2_O_3_ MNPs are widely applied as synergists for halogenated flame retardants in curtains, carpets, plastics, and other products with frequent human contact. The morphology of Sb_2_O_3_ MNPs was characterized by scanning electron microscopy (SEM), with results presented in Figure 1a,b. The Sb_2_O_3_ MNPs adopted polyhedral structures including octahedral, cubic, prismatic, and plate-like morphologies, indicating the coexistence of cubic (α-phase) and orthorhombic (β-phase) crystal systems, and exhibited a broad size distribution from 0.08 to 1.10 μm. The particle size of Sb_2_O_3_ spans both the nanoscale and microscale, which is consistent with the actual size range of Sb_2_O_3_ particles found in nature. However, it should be noted that due to the broad particle size distribution, it is difficult to distinguish between the transformation behavior of nanoscale and microscale Sb MNPs in this study. Elemental composition was further characterized via EDS mapping under scanning transmission electron microscopy (STEM) mode. As shown in Figure 1c,d, intense Sb and O signals were detected throughout the particles, with their spatial distribution matching the particle contours well. To rule out the presence of Sb_2_O_5_, lattice fringe spacing was measured using high-resolution transmission electron microscopy (HRTEM). The measured lattice spacing of 0.327 nm was consistent with the (222) crystal plane of Sb_2_O_3_ (Figure 1f,g), verifying the Sb_2_O_3_ composition of the particles.

Compared with Sb_2_O_3_ MNPs, Sb_2_O_5_ and Sb_2_S_3_ MNPs exhibited more irregular morphologies (Figures S1 and S2). Sb_2_O_5_ MNPs appeared as irregular particles, thin flakes, and elongated rods, with particle sizes ranging from 0.42 to 6.16 μm. Sb_2_S_3_ MNPs had a rougher surface decorated with abundant fine adherent particles, and their overall sizes fell within the range of 0.38–7.01 μm. EDS mapping characterization (Figures S1c–e and S2c–e) detected strong Sb signals in both types of particles, along with intense O and S signals for Sb_2_O_5_ and Sb_2_S_3_, respectively, verifying their elemental compositions. Similarly, lattice fringe spacings of Sb_2_O_5_ and Sb_2_S_3_ MNPs were measured via HRTEM (Figures S1g and S2g), giving values of 0.316 nm and 0.262 nm, which matched the (311) and (131) crystal planes of the respective phases.

The transformation of Sb MNPs involves multiple antimony species, including particulate Sb, Sb(III), and Sb(V), yet no well-established analytical method is currently available for the simultaneous separation and quantification of these distinct Sb forms. To accurately characterize the transformation behavior of Sb MNPs, development of a reliable speciation analysis method is an essential prerequisite.

**Figure 2:**
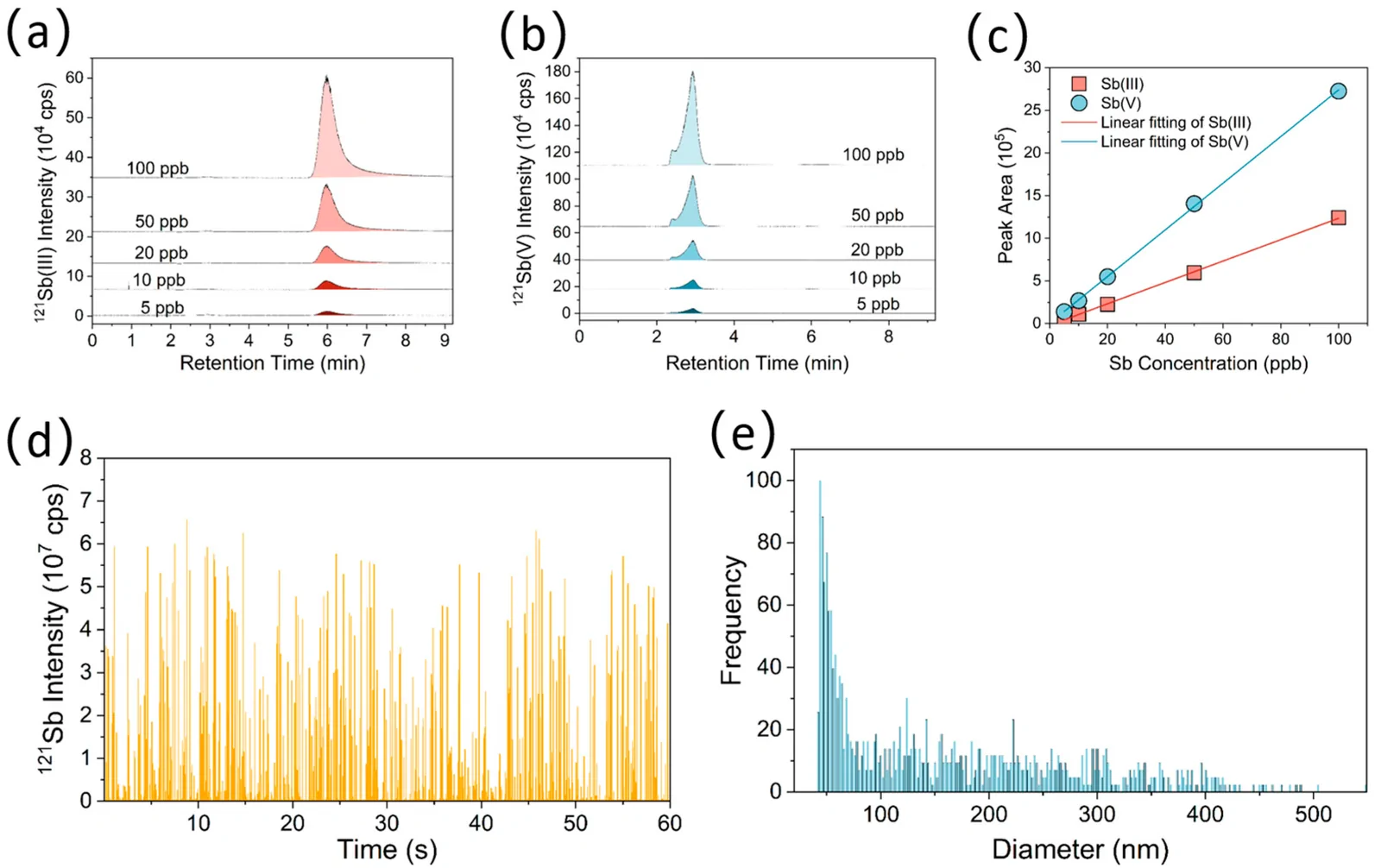
Identification and quantification of different Sb species by LC-ICP-MS and spICP-MS. (**a**,**b**) are LC-ICP-MS chromatograms of Sb(III) and Sb(V), respectively. (**c**) Linear fitting of Sb concentration to the corresponding peak area obtained by LC-ICP-MS. (**d**,**e**) Quantification of particulate Sb by spICP-MS.

To this end, a method for the separation and quantification of Sb(III) and Sb(V) based on LC-ICP-MS was established. It is worth noting that, although the ionic Sb fraction was collected by filtration through a 0.22 µm membrane, which may leave small-sized particulate Sb in the filtrate, LC-ICP-MS can only measure Sb(III) and Sb(V), thus eliminating any contribution from this particulate Sb. As shown in Figure 2, the two Sb oxidation states achieved complete baseline separation, and both exhibited excellent linearity (R^2^ > 0.999) across the spiked concentration range of 5–100 μg/L. As summarized in Table S3, the limits of detection were 0.14 μg/L for Sb(III) and 0.05 μg/L for Sb(V), which fully satisfy routine analytical requirements. Notably, membrane filtration was integrated into the method to remove macromolecular matrix components and matrix-bound Sb species, such that only free Sb targets in the filtrate were quantified. This focus on free Sb species carries clear toxicological significance. Converging evidence shows that contaminants bound to biological matrices exert far lower bioaccessibility than their free counterparts, and it is the free fraction that typically drives pronounced bioaccessibility. Accordingly, our investigation of Sb ion transformation focuses on the behavior of free Sb ions of different valence states.

On the other hand, an sp-ICP-MS method and corresponding pretreatment protocol were established to quantify the particle number concentration of Sb MNPs. Sb_2_O_3_ MNPs were spiked into simulated colonic fluid and extracted via ultrasound-assisted extraction; after quiescent settling, the particle number concentration of target Sb MNPs was directly determined by sp-ICP-MS. It is worth noting that the Sb MNPs recovered by ultrasound-assisted extraction represent only the extractable fraction of the spiked particles, rather than the total amount. Moreover, processes such as aggregation and sedimentation may occur in the simulated fluids, thereby altering the particle concentration. As shown in Figure 2d,e, the method clearly distinguished pulse signals of target particles from background noise, confirming its detection capability for Sb MNPs. Additionally, the size limit of detection, calculated based on the elemental response of antimony, was approximately 42 nm, which adequately meets most analytical requirements. Notably, Sb-containing particles recovered after ultrasound-assisted extraction are regarded as either free particles or particles loosely bound to the biological matrix, exhibiting a degree of bioavailability and worthy of investigation. By contrast, unextracted Sb particles tightly bound to the matrix exert lower bioaccessibility and were not the primary focus of this study.

### 3.2. Transformation Kinetics of Sb MNPs in Typical Simulated Body Fluids

**Figure 3:**
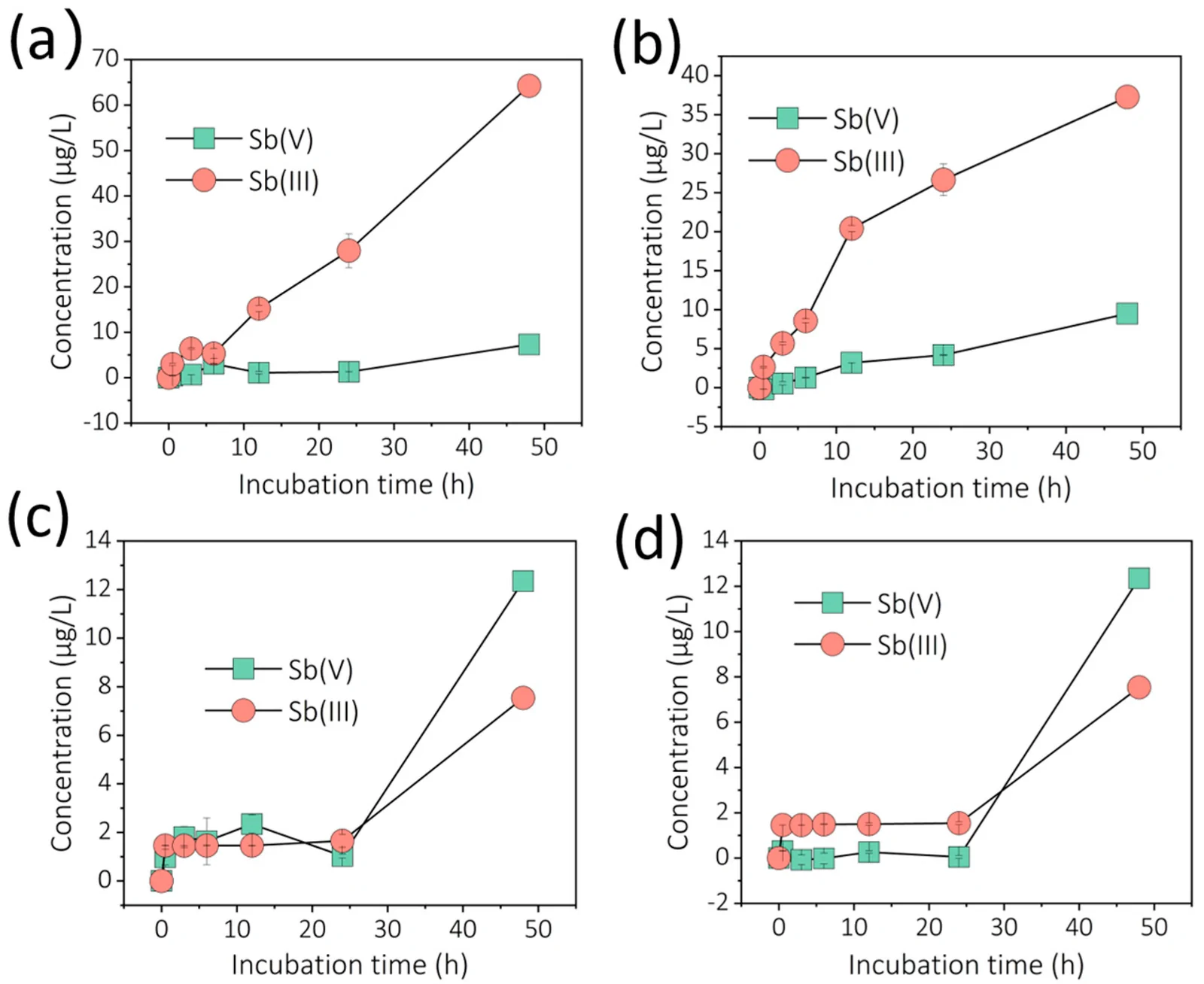
Dissolution kinetics of Sb_2_O_3_ MNPs in simulated saliva (SS) (**a**), simulated gastric fluid (SGF) (**b**), simulated small intestinal fluid (SSIF) (**c**), and simulated colonic fluid (SCF) (**d**).

Given the three major exposure routes of Sb (ingestion, inhalation, and dermal contact), it is essential to clarify the transformation kinetics of representative Sb MNPs in each simulated body fluid and examine how different matrix types modulate their transformation behavior. Sb_2_O_3_ MNPs, the most widely applied Sb species, were spiked into individual simulated fluids at a concentration of 2 mg Sb/L. Although the single concentration cannot cover the range found in real environments, the primary aim of this study was to investigate the transformation behavior of Sb MNPs, which is governed mainly by their physicochemical properties and environmental factors such as pH, temperature, and the composition of the simulated fluids. Therefore, a single representative concentration was selected for this study. Their transformation kinetics were monitored at designated incubation time points in SS, SG, SSIF, and SCF. The ion release kinetics are presented in Figure 3. Across all compartments of the simulated digestive system, free Sb ion concentrations remained generally low, never exceeding 70 μg/L. In SS and SG specifically, the concentration of the more toxic Sb(III) showed a relatively pronounced and continuous upward trend. In contrast, in SSIF and SCF, free Sb levels were extremely low (< 5 μg/L) during the first 24 h of exposure, but increased markedly at 48 h, particularly for Sb(V). A possible explanation is that prolonged incubation in intestinal fluids allows metal-complexing functional groups from proteases and other matrix constituents to gradually erode the surface structure of Sb MNPs. Once the particle surface is breached, internal Sb ions are rapidly released via complexation and subsequently oxidized to higher-valence Sb(V) by dissolved oxygen and oxidative biomolecules in the matrix. As Sb(III) is well documented to be far more toxic than Sb(V), it can be inferred that Sb_2_O_3_ MNPs exert greater bioaccessibility in saliva and gastric fluid than in small intestinal and colonic fluids under single-compartment exposure.

**Figure 4:**
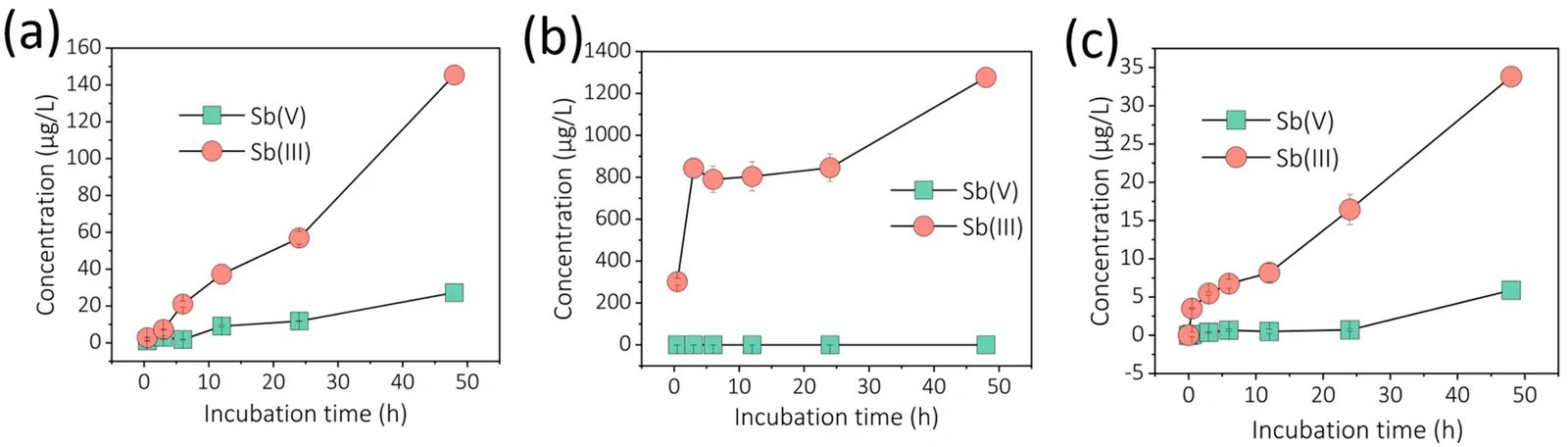
Dissolution kinetics of Sb_2_O_3_ MNPs in Gamble’s solution (GS) (**a**), artificial lysosomal fluid (ALF) (**b**) and simulated sweat (SW) (**c**).

In addition to the simulated digestive ingestion exposure, the ion release kinetics of Sb_2_O_3_ MNPs (spiked at 2 mg Sb/L) were further characterized in simulated lung fluids, i.e., Gamble’s solution (GS) and artificial lysosomal fluid (ALF), for the inhalation pathway, and in simulated sweat (SW) for the dermal contact pathway, as illustrated in Figure 4. In simulated sweat, although the concentration of released Sb(III) rose continuously, its maximum level at 48 h post-exposure was only ~35 μg/L, accounting for 1.75% of the total Sb mass, indicating a low acute exposure of ionic Sb via dermal exposure. By contrast, Sb_2_O_3_ MNPs exhibited markedly higher lability in simulated lung fluids, with substantially elevated Sb(III) release. In ALF in particular, dissolved Sb(III) concentrations increased rapidly within a short timeframe, reaching 1276.7 μg/L at 48 h post-exposure and corresponding to an ion release efficiency of 63.8% of the total exposed Sb. This suggests that Sb_2_O_3_ MNPs may exert stronger influence in ALF. Two underlying mechanisms might drive this phenomenon. First, the low pH of ALF promotes the partial dissolution of Sb_2_O_3_ MNPs and the release of Sb(III). Second, ligands abundant in ALF, such as citrate, lactate, and Cl^−^, readily sequester the newly liberated Sb^3+^ to form stable soluble complexes, which greatly facilitates the sustained dissolution of Sb_2_O_3_. Taken together, these findings further indicate that the transformation of Sb_2_O_3_ MNPs is more pronounced in simulated respiratory fluids.

**Figure 5:**
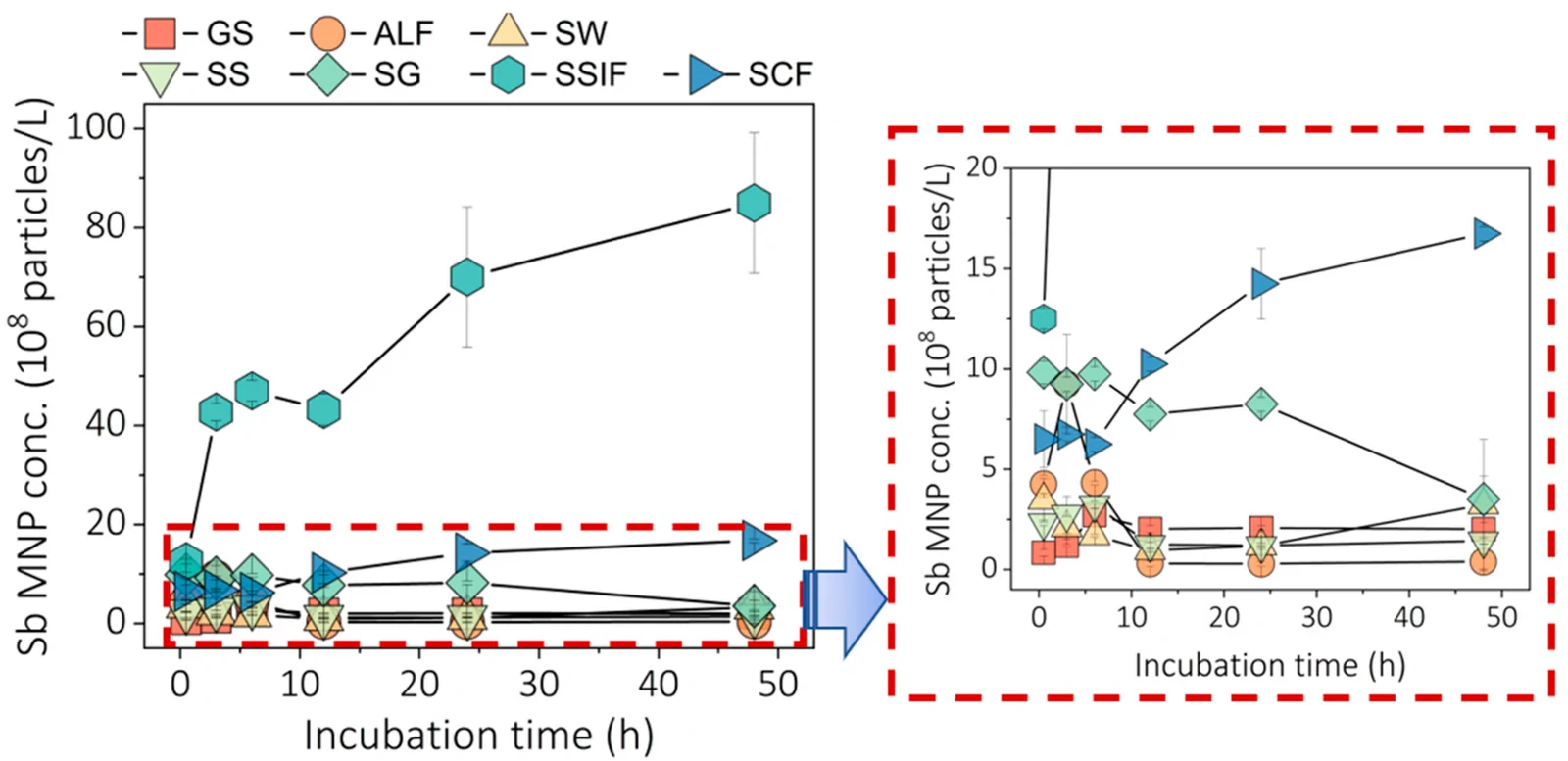
Kinetics of particulate transformation products of Sb_2_O_3_ MNPs in simulated digestive fluids (simulated saliva (SS), simulated gastric fluid (SGF), simulated small intestinal fluid (SSIF), simulated colonic fluid (SCF)), simulated lung fluids (Gamble’s solution (GS) and artificial lysosomal fluid (ALF)), and simulated sweat (SW).

Beyond the release kinetics of Sb ions of different oxidation states from Sb_2_O_3_ MNPs, the dynamic changes in the number concentration of Sb-containing particles across various simulated body fluids were also quantitatively monitored, as presented in Figure 5. The number concentration of free Sb-containing particles in simulated small intestinal fluid was notably higher than in all other tested fluids, and showed a general upward trend with increasing exposure duration. This observation may arise from two mechanisms: first, complexed Sb ions formed by the binding of Sb to metal-coordinating organic functional groups in the simulated biological matrix undergo partial reprecipitation into particulate forms under weakly alkaline conditions; second, abundant inorganic salts and proteases in intestinal fluid can trigger the gradual release of Sb particles adsorbed onto the reactor walls via salting-out effects and competitive adsorption. By contrast, the number concentrations of Sb-containing particles in the remaining exposure groups remained consistently low. This is presumably due to partial particle dissolution driven by the low-pH environment in some simulated fluids, as well as the adsorption and subsequent sedimentation of Sb particles by solid matrix components in other fluids such as simulated colonic fluid, which keeps the abundance of free Sb MNPs at a persistently low level.

### 3.3. Transformation Behavior of Sb MNPs in a Simulated Human Intestinal Microbiota Ecosystem (SHIME)

In realistic human ingestion exposure, ingested Sb MNPs first interact with oral saliva in their pristine form, and the products incubated in oral fluids then pass sequentially through gastric fluid before reaching the small intestinal and colonic compartments. For each simulated digestive fluid, the Sb species to which it is exposed differ from the initially administered Sb MNPs. Accordingly, it is necessary to characterize the transformation behavior of Sb MNPs across the full digestive tract following oral ingestion. To this end, a dynamic simulated human intestinal microbiota ecosystem was first established. The system was configured with comprehensive physiological parameters, including oxygen tension, system pH, and human fecal suspension to faithfully recapitulate the native human gut microbial community.

**Figure 6:**
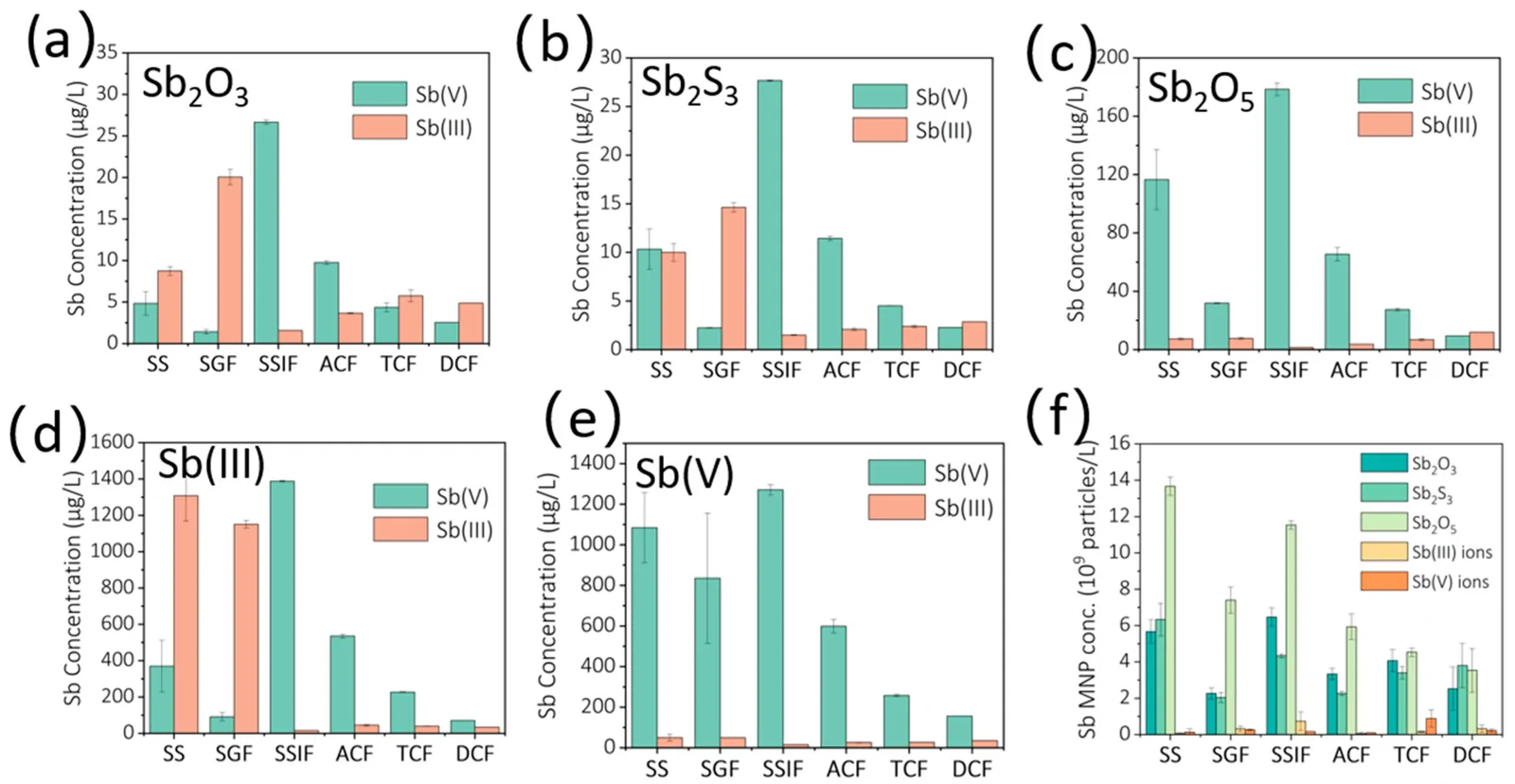
Transformation products of Sb MNPs and ionic Sb species in the SHIME system. (**a**–**e**) Sb(III) and Sb(V) concentration after exposure of Sb_2_O_3_, Sb_2_S_3_, Sb_2_O_5_, Sb(III), and Sb(V). (**f**) Sb MNPs concentration detected by sp-ICP-MS after exposure of different Sb species.

To represent the dominant chemical forms of Sb in the natural environment, this study characterized the transport and transformation of three Sb MNPs (Sb_2_O_3_, Sb_2_S_3_, and Sb_2_O_5_) and two ionic Sb species (Sb(III) and Sb(V)) across the full digestive tract. It should be noted that the above Sb-containing particles investigated in this study are not exclusively nanosized, and a substantial fraction of the particles fall within the micrometer size range. Therefore, the transformation behavior observed in this work cannot be directly and fully extrapolated to the size-specific behavior and risk assessment of pure Sb-based nanomaterials. Transformation processes were quantitatively monitored using the LC-ICP-MS and sp-ICP-MS methods established above. Each of the five Sb target species was spiked into simulated saliva at 2 mg Sb/L. In accordance with the physiological transit patterns and in vivo residence times of food in the human digestive system, samples were sequentially incubated in simulated gastric fluid, small intestinal fluid, ascending colon fluid, transverse colon fluid, and descending colon fluid. Samples were retrieved from each simulated digestive compartment to track the transformation of target species. As illustrated in Figure 6, simulated gastric fluid induced partial dissolution of Sb_2_O_3_, Sb_2_S_3_, and Sb_2_O_5_ MNPs into their corresponding ionic forms, with no change in the original valence state of the dissolved Sb. In the small intestinal phase, however, most of the Sb(III) generated in the gastric compartment was oxidized to Sb(V). Although further evidence is required, a tentative explanation is that the oxidation may be mediated by multiple factors—dissolved oxygen from ingested water, redox-active biomolecules (e.g., bilirubin) in small intestinal fluid, and abundant endogenous enzymes—all of which could promote the conversion of Sb(III) to Sb(V). Given that Sb(V) is considerably less toxic than Sb(III), the Sb(III)-to-Sb(V) transition in the small intestine facilitates conversion to a less toxic oxidation state. In the ascending, transverse, and descending colon phases, concentrations of both Sb(III) and Sb(V) declined progressively. This trend is probably due to the higher content of solid fractions in colonic fluid relative to other simulated fluids. A fraction of Sb ions binds to the surfaces of these solid components and is retained by membrane filters during sample pretreatment, leading to a decrease in bioavailable free ionic Sb. This result further indicates that the colonic phase also contributes to the partial conversion of Sb to a less toxic oxidation state. Notably, the altered Sb speciation and release behavior observed in the SHIME system indicate that gut microbiota may be involved in the transformation of Sb species. However, given that the specific contribution of the microbiota was not independently assessed in this work, the microbiota-mediated transformation pathway proposed herein remains a potential mechanism that has not been directly validated.

In addition, sp-ICP-MS was used to monitor variations in the number concentration of Sb MNPs along the simulated digestive tract, as shown in Figure 6f. Across all three Sb particle exposure groups, the highest number concentration of Sb-containing particles was detected in the initial salivary phase. The low-pH gastric environment caused a significant drop in particle number concentration, especially for the most ubiquitous Sb_2_O_3_ particles, as acidic conditions promote particle dissolution. This trend is consistent with the ionic concentration profiles obtained via LC-ICP-MS. Compared with gastric fluid, small intestinal fluid contained a markedly higher number of Sb MNPs. This increase is mainly attributed to the higher pH value of the intestinal environment, which drives the gradual precipitation of Sb ions and their reformation into particulate matter. In the colonic phase, the number of Sb-containing particles decreased substantially. The underlying mechanism is likely analogous to that for the reduction in ionic Sb: complex solid matrix components in colonic fluid reduce the abundance of free Sb MNPs through adsorptive binding of the target species.

## 4. Conclusions

In this study, in vitro models mimicking human gastrointestinal, respiratory, and dermal exposure pathways were established to quantitatively elucidate the transformation behaviors and underlying mechanisms of representative Sb MNPs in human-relevant biological media. The results demonstrate that the transformation of Sb MNPs is markedly organ-dependent. In simulated gastric fluid, Sb_2_O_3_, Sb_2_S_3_, and Sb_2_O_5_ all dissolved into ionic forms with no alteration in their oxidation states. In the intestinal phase, by contrast, Sb(III) was oxidized to the less toxic Sb(V). Dermal sweat exhibited extremely weak dissolution capacity toward Sb MNPs, with an ion release rate below 1.75%, indicating a relatively low risk via dermal exposure. However, Sb_2_O_3_ underwent pronounced dynamic dissolution in simulated lung fluids, especially in ALF, achieving an ion release rate as high as 63.8%. This phenomenon is mainly ascribed to the low-pH environment and the complexation-promoting effect of organic ligands, suggesting that simulated respiratory fluids serve as an important site for the transformation of Sb MNPs. Although this study systematically investigated the transformation of Sb MNPs in various typical simulated body fluids, certain limitations remain, including the simplified nature of the in vitro systems, the use of a single exposure concentration, and the lack of direct toxicological measurements. We hope that our work may provide inspiration and methodological support for studying the transformation of Sb MNPs in human biological media.

## Data Availability

The raw data supporting the conclusions of this article will be made available by the authors on request.

## Supplementary Materials

The following supporting information can be downloaded at: https://www.mdpi.com/article/10.3390/nano16160974/s1, including formulas of Simulated Body Fluids, instrument parameters, analytical performance for Sb(III) and Sb(V) by LC-ICP-MS, characterization of Sb_2_O_5_ and Sb_2_S_3_ MNPs. Figure S1: Characterization of Sb_2_O_5_ MNPs; Figure S2: Characterization of Sb_2_S_3_ MNPs; Table S1: Instrumental and analytical details for Sb(III) and Sb(V) by LC-ICP-MS; Table S2: Instrumental and analytical details for Sb MNPs by sp-ICP-MS; Table S3: Method performance for Sb(III) and Sb(V) by LC-ICP-MS.
