# Supplementary material for: Quantitative Investigation of the Fate and Behavior of Antimony Micro/Nanoparticles in Simulated Body Fluids
Source: Nanomaterials (Basel). 2026 Aug 7;16(16):974. doi: 10.3390/nano16160974 (PMC13515899; doi:10.3390/nano16160974)
Supplement: Supplementary file 1 [file nanomaterials-16-00974-s001.zip › nanomaterials-4439113-supplementary.pdf]

Supporting Information for

Quantitative Investigation of the Fate and Behavior of Antimony Micro/Nano-Particles in Simulated Body Fluids

Yujian Lai[1], Sujuan Yu[2], Zhensong zhang[2], and Lijie Dong[3,*]

[1]Department of Environmental Science and Engineering, School of Energy and Environmental Engineering, The University of Science and Technology Beijing, Beijing 100083, China
[2]Research Center for Eco-Environmental Sciences, Chinese Academy of Sciences, P.O. Box 2871, Beijing 100085, China
[3]Division of Chemical Metrology and Analytical Science, National Institute of Metrology, China, Beijing 100029, China

* Corresponding author
E-mail: donglj@nim.ac.cn

Text S1. *In Vitro* Gastrointestinal Digestion Model

An *in vitro* gastrointestinal digestion model was developed based on established protocols to replicate the full transit of ingested Sb MNPs alongside food matrix from the oral cavity through the stomach and intestinal tracts. The model comprised six independent glass vessels corresponding to the oral cavity, stomach, small intestine, ascending colon, transverse colon, and descending colon, respectively. Each vessel was fitted with a glass-coated magnetic stir bar and agitated at a constant speed to mimic oral mastication and gastrointestinal peristalsis, with the system temperature maintained at 37 °C throughout. Given that food retention times vary across digestive organs and digestion does not initiate simultaneously in all compartments, the vessels were not directly connected; instead, the digested mixture was manually transferred between successive compartments. During operation, the pH of each simulated digestive fluid was monitored periodically and fine-tuned with 0.5 mol/L HCl or 0.5 mol/L NaOH to maintain near-constant pH in each compartment, closely reproducing the *in vivo* gastrointestinal environment. All vessels were wrapped in aluminum foil to simulate the dark conditions of internal human organs. Additionally, all apparatus used in the procedure, as well as all pH monitoring and fluid transfer operations, were conducted under strictly aseptic conditions.

The detailed operating procedure was as follows. First, 2 g of salivary amylase was added to 200 mL of simulated food matrix. After 5 min of homogeneous stirring, the mixture was transferred to the gastric vessel, mixed with 20 mL of gastric acid solution, and incubated at pH 3.1 for 2 h. The digesta was then transferred to the small intestinal vessel, combined with 50 mL of pancreatic juice solution, and incubated at pH 7.1 for 4 h before being delivered to the colonic compartments. Prior to the colonic phase, 225 mL of simulated food matrix and 25 mL of human fecal slurry were added to each of the ascending, transverse, and descending colon vessels, and homogenized at 37 °C for 24

h to allow microbial stabilization. Once the colonic mixtures and microbial communities were stabilized, the digesta from the small intestine was introduced into the ascending colon vessel. After 12 h of incubation, the mixture was transferred to the transverse colon vessel for a further 12 h, and finally moved to the descending colon vessel for an additional 12 h to complete the full simulated digestion process. All incubation steps were performed at 37 °C with continuous stirring.

Detailed preparation procedures for all simulated digestive fluids involved in the digestive system model are provided below.

Simulated Food Fluid (SFF): Prepared in accordance with published protocols. The following components were dissolved in 1 L of deionized water: 1 g arabinogalactan, 2 g pectin, 1 g xylan, 3 g potato starch, 0.4 g glucose, 3 g yeast extract, 1 g special peptone, and 0.5 g cysteine. The resulting solution was autoclaved at 121 °C for 15 min, cooled to room temperature, and stored at 4 °C until use.

Simulated Human Saliva (SS): The following reagents were sequentially dissolved in deionized water: 1117 mg/L KCl, 503 mg/L KH_2PO_4, 1142 mg/L $NaHCO_3$, 144 mg/L $MgCl_2·6H_2O$, 5.76 mg/L $(NH_4)_2CO_3$, 1257 mg/L $CaCl_2·2H_2O$, 91 mL/L concentrated hydrochloric acid, and 833 mg/L α-amylase. The mixture was thoroughly stirred to homogeneity to yield simulated human saliva, which was stored at 4 °C prior to use.

Simulated Gastric Fluid (SGF): SGF was prepared by mixing simulated gastric acid (SGA) with SFF at a volume ratio of 1:10, followed by pH adjustment to 3.1. The SGA stock solution was formulated by dissolving 3.2 g of porcine pepsin (Aladdin, Shanghai), 2 g of NaCl, and 100 mL of concentrated hydrochloric acid in deionized water and diluting to a final volume of 1000 mL, with the pH adjusted to 1.1. The freshly prepared SGF was stored at 4 °C until use.

Simulated Small Intestinal Fluid (SSIF): SSIF was prepared by mixing simulated pancreatic juice (SPJ) with SGF at a volume ratio of 1:4.4, with the pH adjusted to 7.1. The SPJ stock solution was prepared by dissolving 12.5 g of $NaHCO_3$, 6 g of bovine bile, and 0.9 g of pancreatin (Aladdin, Shanghai) in deionized water and diluting to 1000 mL. The final SSIF mixture was stored at 4 °C until use.

Simulated Colonic Fluid (SCF): SCF was prepared by mixing fecal slurry with SSIF at a volume ratio of 1:10, followed by pH adjustment to 6.1. The fecal slurry was prepared as follows. Fresh fecal samples were collected from five healthy adults who had not received antibiotics in the preceding 6 months and pooled thoroughly. The pooled fecal mixture was added to sodium thioglycolate-phosphate buffer at a mass-to-volume ratio of 1:5; the buffer was prepared by dissolving 1 g of sodium thioglycolate in 1 L of phosphate buffer. After homogenization, the sample was transferred to a 1 L glass bottle, stirred for 3 min, and allowed to stand for 10 min, followed by centrifugation at 1000 rpm and 4 °C for 10 min to collect the supernatant. Approximately 30 mL of the clarified supernatant was transferred to centrifuge tubes, mixed thoroughly with 7 mL of glycerol, and stored at −80 °C.

Text S2. *In Vitro* Respiratory Model

In vitro respiratory tract exposure models typically employ two simulated fluid systems: Gamble's solution (GS) and artificial lysosomal fluid (ALF). GS mimics the interstitial lining fluid of the deep lung, whereas ALF represents the fluid environment encountered by fine particles after endocytosis by alveolar and interstitial macrophages. Specifically, ALF was prepared by dissolving the following components in deionized water: 107 mg/L $MgCl_2 \cdot 6H_2O$, 3210 mg/L NaCl, 179 mg/L $Na_2HPO_4 \cdot 12H_2O$, 39 mg/L Na_2SO_4, 128 mg/L $CaCl_2 \cdot 2H_2O$, 77 mg/L trisodium citrate dihydrate ($C_6H_5Na_3O_7 \cdot 2H_2O$), 6000 mL/L NaOH, 22700 mg/L citric acid, 59 mg/L glycine, 90 mg/L sodium tartrate, 85 mg/L sodium lactate, 86 mg/L sodium pyruvate, and 50 mg/L benzalkonium chloride. After thorough mixing, the pH of the solution was adjusted to 4.5. GS was prepared by dissolving the following reagents in deionized water: 203 mg/L $MgCl_2 \cdot 6H_2O$, 6020 mg/L NaCl, 298 mg/L KCl, 318 mg/L $Na_2HPO_4 \cdot 12H_2O$, 63 mg/L Na_2SO_4, 368 mg/L $CaCl_2 \cdot 2H_2O$, 952 mg/L sodium acetate, 2604 mg/L $NaHCO_3$, 97 mg/L trisodium citrate dihydrate ($C_6H_5Na_3O_7 \cdot 2H_2O$), and 50 mg/L benzalkonium chloride. The solution was fully homogenized under stirring and adjusted to pH 7.4. Both prepared ALF and GS solutions were stored at 4 °C until use.

Text S3. *In Vitro* Sweat Model

Following previously reported protocols, simulated sweat (SW) was prepared by dissolving sodium chloride, urea, and lactic acid in deionized water to final concentrations of 5.0, 1.0, and 1.0 g/L, respectively. The solution was thoroughly homogenized under stirring and adjusted to pH 6.5. The prepared SW solution was stored at 4 °C prior to use.

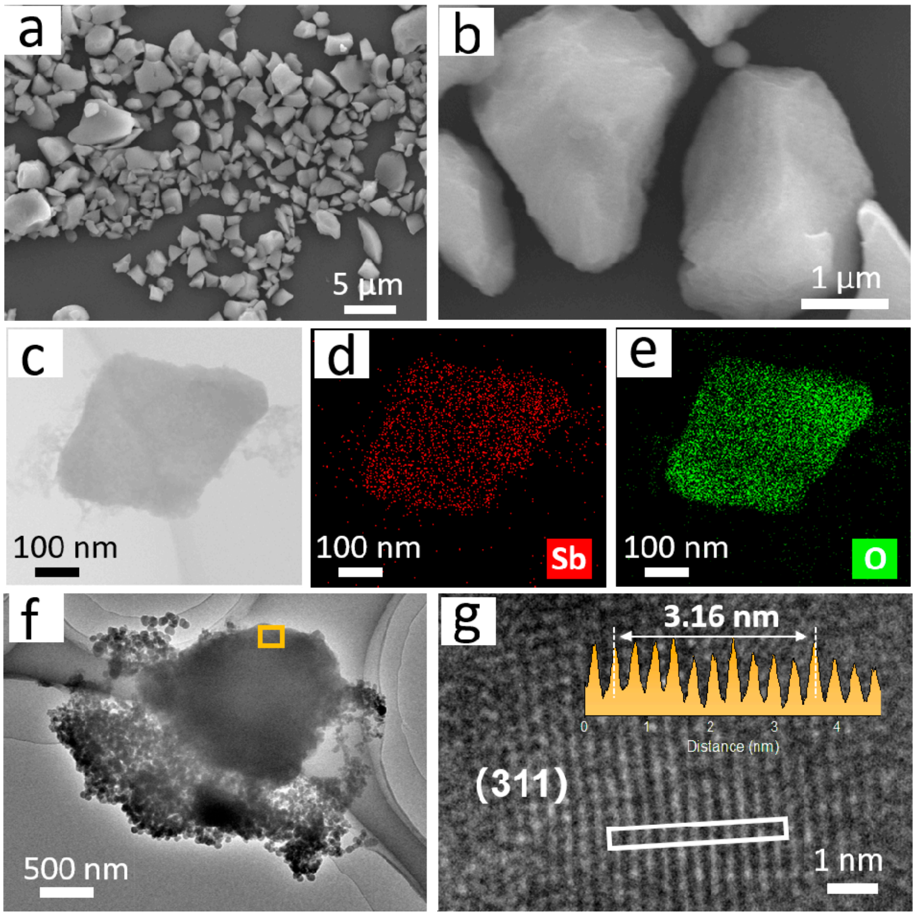

Figure S1. Characterization of Sb_2O_5 MNPs. (a) Low-magnification SEM image; (b) high-magnification SEM image; (c) STEM image; (d, e) EDS elemental mapping images of Sb and O, respectively; (f) HRTEM image; (g) lattice fringes from HRTEM characterization in the rectangular area of figure (f).

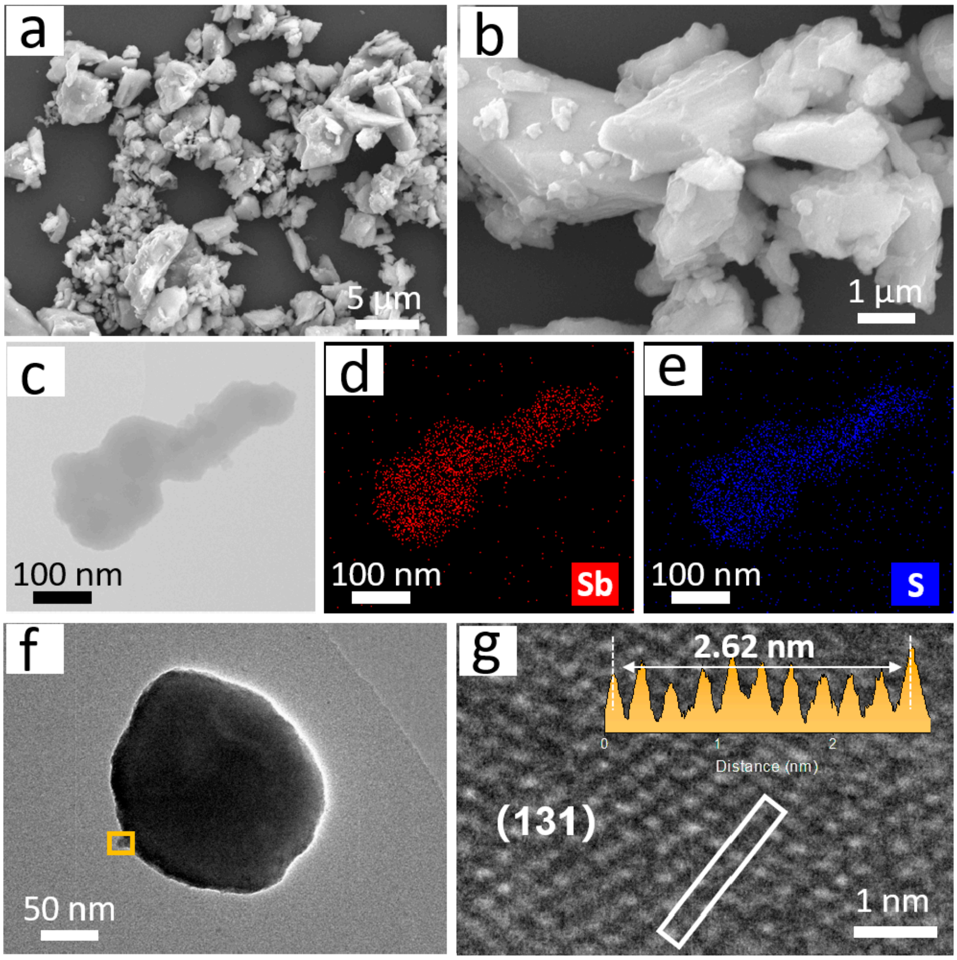

Figure S2. Characterization of Sb_2S_3 MNPs. (a) Low-magnification SEM image; (b) high-magnification SEM image; (c) STEM image; (d, e) EDS elemental mapping images of Sb and S, respectively; (f) HRTEM image; (g) lattice fringes from HRTEM characterization in the rectangular area of figure (f).

Table S1. Instrumental and analytical details for Sb(III) and Sb(V) by LC-ICP-MS.

Parameter group	Parameter	Parameter value
ICP-MS component	ICP-MS model	Agilent 8900 Triple Quad
	Nebulizer	MicroMist, glass
	Spray chamber	Quartz
ICP-MS settings	RF power	1550 W
	Nebulizer gas flow	1.02 L/min
	Dwell time	0.1 s
	Signal collection mode	TRA
	Reaction gas	He
SEC parameters	LC model	Agilent 1200 series
	Column	Hamilton PRP-X100, 10 μm, 250 mm× 4.1 mm
	Column temperature	25 °C
	Injection volume	20 μL
	Flow rate	1.0 mL/min
	Mobile phase	25 mM sodium tartrate, 10 mM EDTA, 80 mM NH_4NO_3

Table S2. Instrumental and analytical details for Sb MNPs by sp-ICP-MS.

Parameter group	Parameter	Parameter value
ICP-MS component	ICP-MS model	Agilent 8900 Triple Quad
	Nebulizer	MicroMist, glass
	Spray chamber	Quartz
ICP-MS settings	RF power	1550 W
	Nebulizer gas flow	1.01 L/min
	Sample flow rate	0.270 mL/min
	Dwell time	0.1 s
	Total acquisition time	60 s
	Reaction gas	He

Table S3. Method performance for Sb(III) and Sb(V) by LC-ICP-MS.

Analytes	Retention Time (min)	Linear Range (µg/L)	Correlation coefficients (R^2)	LOD (µg/L)	LOQ (µg/L)	Sensitivity	Recovery (%)
Sb(III)	5.98	5-100	0.9996	0.14	0.48	27332	80.7
Sb(V)	2.93	5-100	0.9996	0.05	0.15	12528	78.5
